# Finite element evaluation of a novel adjustable-length partial ossicular replacement prosthesis

**DOI:** 10.3389/fbioe.2026.1956560

**Published:** 2026-10-12

**Authors:** Qingsong Liu, Xiao Qin, Songbo Xue, Zhiqiang Gao, Guodong Feng

**Affiliations:** 1 Department of Otolaryngology, Peking Union Medical College Hospital, Chinese Academy of Medical Sciences, Beijing, China; 2 Department of Skull Base Surgery Center, Huanhu Hospital Affiliated to Tianjin Medical University, Tianjin, China

**Keywords:** conductive hearing loss, finite element analysis (FEA), partial ossicular replacement prosthesis (PORP), adjustable length, polyetheretherketone (PEEK)

## Abstract

**Purpose:**

Partial ossicular replacement prosthesis (PORP) reconstruction is a standard otologic procedure for restoring sound conduction in patients with conductive hearing loss resulting from partial ossicular chain disruption. Current commercial PORP designs exhibit several limitations that may affect long-term hearing outcomes and contribute to complication risks. This study presents a novel adjustable-length PORP and systematically evaluates its biomechanical performance and sound transmission fidelity using a finite element analysis (FEA) framework, thereby providing computational evidence for preclinical feasibility evaluation.

**Methods:**

The proposed prosthesis incorporates a deformable bent titanium alloy connecting rod sandwiched between two polyetheretherketone (PEEK) coupling components: a tympanic-side circular disc and a stapes-head bowl. The connecting rod incorporates three independently adjustable bend angles (α, β, γ) that enable intraoperative alignment optimization. An FEA model of the intact human middle ear was constructed, and baseline vibratory transmission characteristics were quantified using the stapes footplate frequency response curve. An FEA model of the middle ear reconstructed with the novel adjustable-length PORP was then generated, and vibratory conduction performance was quantified across continuous length settings spanning 2.3–3.5 mm.

**Results:**

Across the evaluated length range of 2.4–3.5 mm, the prosthesis demonstrates consistent stapes footplate velocity transmission, with predicted hearing loss remaining below 17 dB across the 100 Hz–10 kHz frequency range. Adjusting the prosthesis to the minimum 2.3 mm length increased maximum hearing loss to 34 dB, consistent with structural overcompression.

**Conclusion:**

This finite element analysis provides computational evidence which suggesting the preclinical biomechanical feasibility of the proposed adjustable-length PORP for ossicular chain reconstruction. The predicted results suggest potentially acceptable sound transmission performance and favorable mechanical conditions at the prosthesis-bone interfaces. These findings warrant further experimental validation using physical middle ear models and animal studies to assess long-term safety and functional outcomes.

## 1 Introduction

Disruption of the ossicular chain is a major cause of conductive hearing loss (Esser and Lüers, 2025; Young and Ng, 2026). PORPs are the standard first-line surgical reconstruction intervention for patients with an intact stapes superstructure (Wang et al., 2024; Young and Ng, 2026). Contemporary PORP implantation can achieve postoperative air-bone gaps (ABG) within 20 dB (Kraus, 1993; Nguyen et al., 2021). Clinical outcomes are known to be modulated by patient-specific anatomy, prosthesis material and structural design, surgical technique, and postoperative care (Govil et al., 2017; Das et al., 2020). A recent meta-analysis of PORP clinical outcomes reports that the benchmark clinical success endpoint of postoperative ABG ≤ 20 dB is achieved in only 70.3% of cases (Canzi et al., 2023; Kortebein et al., 2023), indicating that approximately 30% of patients do not achieve this benchmark.

Several factors limit current PORP outcomes. First, intraoperative length adjustment often requires irreversible trimming of the prosthesis shaft. Second, the substantial stiffness mismatch between titanium prostheses (elastic modulus ∼110 GPa) and native ossicular bone (10–20 GPa) contributes to stress shielding. Third, no widely adopted PORP design currently offers reversible, continuous length adjustment across the full range of anatomical variation. Iterative optimization of PORP structural design and material properties represents a clear evidence-based pathway to improve postoperative success rates.

Titanium alloy is the most widely used PORP material in current clinical practice (Govil et al., 2017; Faramarzi et al., 2023). The elastic modulus of titanium alloy is approximately 110 GPa (Jia et al., 2024), while the elastic modulus of native human ossicular bone ranges from only 10 to 20 GPa (Areias et al., 2016; Chang et al., 2016). This substantial stiffness mismatch produces severe inhomogeneous mechanical conduction at the prosthesis-bone interface. High-stiffness titanium prostheses bear nearly all transmitted load, while surrounding bone tissue receives insufficient physiological mechanical stimulation, leading to established stress shielding effects (Wang et al., 2023; Sergienko et al., 2025). This pathophysiological process is directly associated with recognized postoperative complications including progressive periprosthetic bone resorption, prosthesis extrusion, positional displacement, and long-term degradation of hearing reconstruction outcomes (Canzi et al., 2023). Large multicenter studies have reported titanium PORP extrusion rates up to 8.4% and displacement rates up to 4.2%, accompanied by deteriorating audiometric performance during extended follow-up.

A second critical unmet clinical need relates to the cumbersome and irreversible mechanism of intraoperative length adjustment for existing PORP designs (Wolferts et al., 2017). The middle ear cavity is a spatially confined anatomical space with profound interindividual morphological variation, requiring precise prosthesis length calibration to span the gap between the tympanic membrane (or manubrium of the malleus) and the stapes capitulum. Prior biomechanical studies have demonstrated that even minor length deviations (50–200 μm) or excessive preload tension can severely degrade the middle ear transfer function, attenuating low-frequency sound transmission by up to 25 dB (Neudert et al., 2016; Schar et al., 2023). Current surgical workflows rely on manual cutting or clipping of the titanium shaft to achieve the desired prosthesis length. This iterative “trial-and-error” trimming process is inherently irreversible: if a prosthesis is inadvertently cut too short during the first fitting attempt, the entire device must be discarded, generating avoidable material waste and increasing procedural costs. Furthermore, repeated measurement, trial fitting, and trimming steps prolong operative duration, elevate surgical site infection risk, and cannot guarantee optimal acoustic coupling tension. An excessively tight fit amplifies mechanical stress on the annular ligament and tympanic membrane, promoting prosthesis extrusion, while an excessively loose predisposes the device to complete dislocation. Collectively, these limitations identify an urgent unmet clinical need for a next-generation PORP that simultaneously resolves both material and structural deficiencies. An optimal prosthesis would feature an elastic modulus more closely matched to native ossicular tissue to reduce stress shielding, combined with a reversible length-adjustment mechanism enabling precise intraoperative calibration.

Polyetheretherketone (PEEK) exhibits excellent long-term biocompatibility, with an elastic modulus (∼3–4 GPa) that closely approximates the mechanical properties of human cortical bone. These favorable properties have supported widespread clinical translation of PEEK in craniofacial reconstruction, spinal implants, and dental prostheses (Wang et al., 2022; Zheng et al., 2023), suggesting PEEK as a promising candidate for ossicular prostheses, although its application in middle ear reconstruction has received limited clinical investigation. Finite element analysis provides a robust computational framework for simulating the complex vibro-acoustic behavior of the middle ear under both physiological and pathological conditions, enabling quantitative prediction of stapes footplate displacement and velocity across the full audible frequency spectrum in response to varying prosthesis designs, material properties, and surgical configurations (Jiang et al., 2025; Wilken et al., 2025; Muyshondt et al., 2026; Zhou et al., 2026).

This study presents a hybrid length-adjustable PORP combining titanium alloy and PEEK. PEEK serves as the coupling material at the tympanic membrane and stapes head interfaces to mitigate stress shielding, while the titanium alloy connecting rod provides structural integrity and enables reversible length adjustment through controlled bending. The primary intended clinical application scenario is direct contact coupling between the PEEK tympanic-side disc and the medial surface of the tympanic membrane, without interposed soft tissue or cartilage graft, for ossicular reconstruction cases where the malleus handle has been eroded or removed. Finite element analysis (FEA) was used to evaluate the mechanical feasibility of this prosthesis preliminary.

## 2 Materials and methods

### 2.1 Design and characterization of the adjustable-length PORP

This study describes a hybrid adjustable-length PORP constructed from titanium alloy and PEEK (Figure 1). The three-component prosthesis consists of a tympanic-side circular disc, a deformable bent connecting rod, and a bowl-shaped stapes connector. The disc and stapes connector are fabricated from medical-grade PEEK, while the bent central connecting rod is manufactured from Ti-6Al-4V titanium alloy.

**FIGURE 1:**
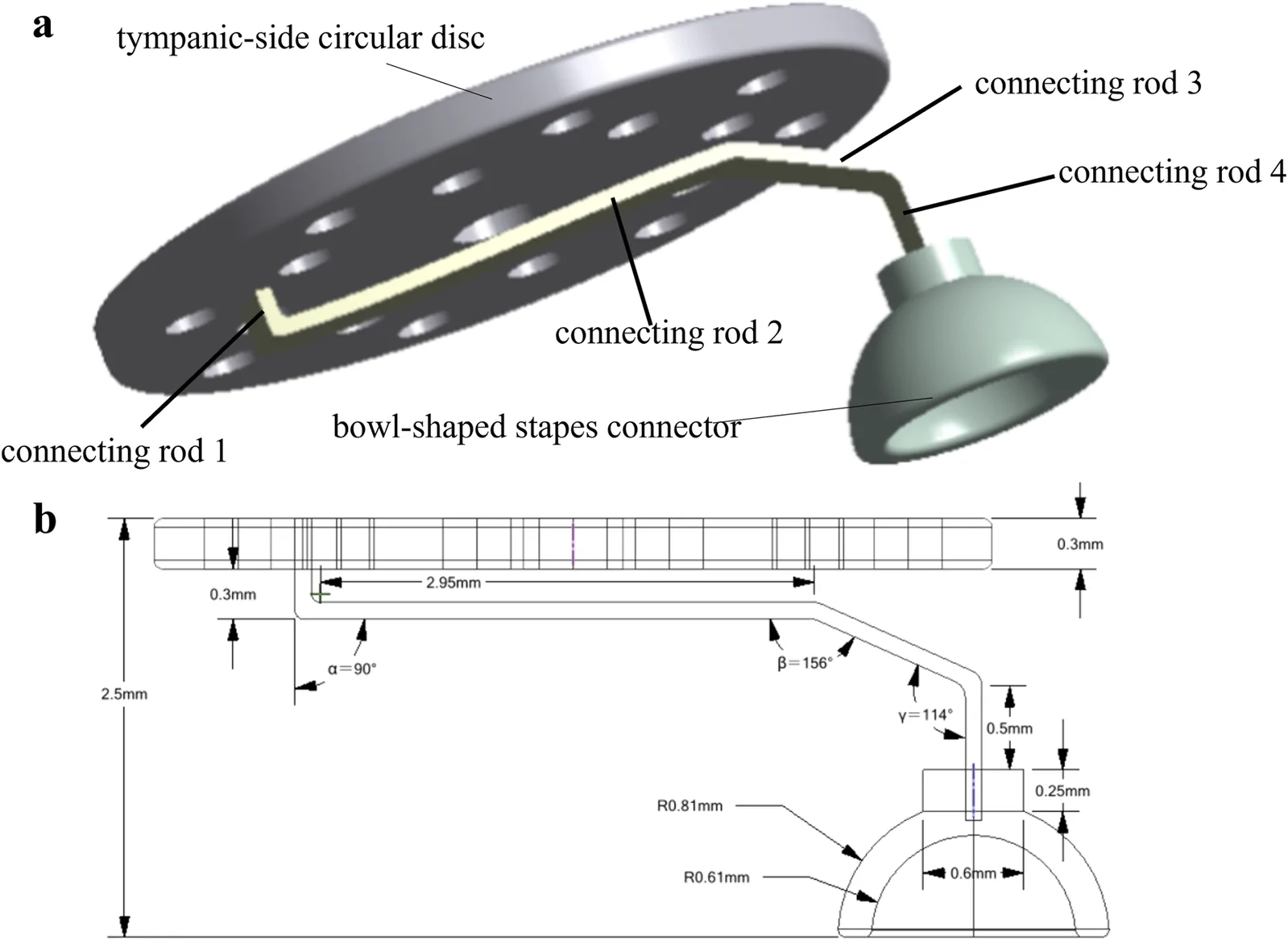
Structural diagram of the novel adjustable-length Partial ossicular replacement prosthesis (PORP). **(a)** Three-dimensional rendering showing the complete PORP assembly including tympanic-side circular disc, connecting rod, and bowl-shaped stapes connector; **(b)** Sectional drawing with dimension annotations.

The bowl-shaped connector is a hemispherical structure with an inner radius of 0.61 mm and a wall thickness of 0.2 mm. Surgeons may further customize the fit by modifying the inner surface of the PEEK connector during surgery. The dimensions were determined based on morphometric studies of adult human stapes anatomy, where the average stapes head diameter ranges from approximately 0.7–0.8 mm. The selected inner radius of 0.61 mm provides appropriate coverage for the majority of adult patients.

The PEEK tympanic-side disc couples directly with the medial surface of the tympanic membrane without interposed cartilage graft, which is the intended clinical configuration for ossicular reconstruction cases where the malleus handle has been eroded or removed by chronic otitis media or cholesteatoma pathology. The elastic modulus of medical-grade PEEK (3–4 GPa) is nearly two orders of magnitude lower than that of conventional titanium alloy (∼110 GPa) and closely matches native human cortical ossicular bone. This drastically reduced interfacial stiffness distributes the transmitted vibratory load evenly across the contact area without inducing excessive localized stress on the tympanic membrane, potentially eliminating the need for cartilage interposition in this design.

Total prosthesis length is precisely calibrated intraoperatively through a two-stage segmented adjustment strategy using the three independent articulation angles (α, β, γ) of the bent titanium connecting rod (Figure 1b). For length adjustment within the 2.3–2.7 mm range, connecting rod two is fixed, and angles β and γ are adjusted to modify the axial projection length of connecting rod 3. For length adjustment beyond 2.7 mm, connecting rod three is fixed, and angles α and β are adjusted to modify the axial projection length of connecting rod 2. The adjustment is performed manually by the surgeon using standard otologic micro-alligator forceps; no specialized custom adjustment device is required. In clinical operation, the total length adjustment can be achieved by adjusting the corresponding angle against a microscope ruler and sequentially adjusting the axial position of connecting rod three at both ends.

To quantitatively evaluate the length adjustability range of the proposed prosthesis, static FEA models with different PORP total prosthesis lengths (defined as the axial distance from the upper surface of the tympanic disc to the distal end of the stapes bowl, initialized at 2.5 mm) were built. Length adjustability was characterized by mapping the prosthesis stress distribution across a continuously adjusted length range from 2.3 to 3.5 mm. All static structural finite element simulations were performed using Abaqus 2022 (Dassault Systèmes, France).

### 2.2 Finite element simulation of normal middle ear sound conduction

The baseline geometric structure of the intact human middle ear was obtained from the publicly available dataset hosted by the Biophysics and Biomedical Physics Research Group of the University of Antwerp^1^. This anatomically accurate model was reconstructed from high-resolution micro-CT scan data of a fresh-frozen cadaveric temporal bone. The full model (Figure 2) includes the tympanic membrane (TM), malleus, incus, stapes, incudomalleolar joint (IMJ), incudostapedial joint (ISJ), tensor tympani muscle (TTM), anterior mallear ligament (AML), lateral mallear ligament (LML), posterior incudal ligament (PIL), stapedial annular ligament (SAL), and stapedius muscle (SM), forming a complete and validated finite element representation of the intact human middle ear. The superior malleal ligament and superior incudal ligament were excluded due to imaging limitations, which cannot adequately resolve these fine soft tissue structures. A 10 mm long external auditory canal model was also constructed to represent physiological acoustic loading conditions. The global mesh element size for the entire model was constrained to ≤0.1 mm to ensure numerical solution accuracy.

**FIGURE 2:**
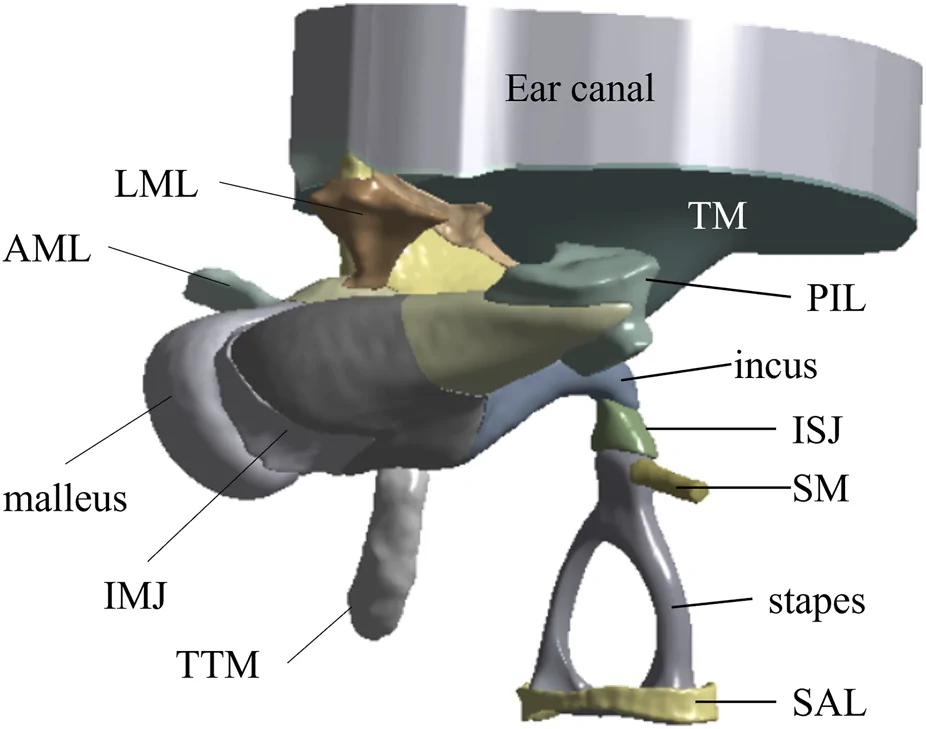
Validated anatomically accurate finite element model of normal human middle ear sound conduction.

Acoustic harmonic response finite element simulations of middle ear vibratory conduction were performed in the ANSYS Workbench 2019 R1 acoustic module. Previous studies have demonstrated that middle ear sound transmission exhibits linear behavior under moderate sound pressure levels (Fay et al., 2006; O’Connor et al., 2017), with maximum strain remaining below 0.1%. Therefore, this study employs a linear elastic material model to describe the dynamic mechanical behavior of the middle ear joints, tendons, and ligaments. Additionally, the parameters of linear elastic materials, including Young’s modulus and Poisson’s ratio, are obtained from literature and cross-calibration (Gan et al., 2004; Zhang et al., 2018; 2020). The assigned elastic modulus (*E*) and density (*ρ*) values are summarized in Table 1. Physiologically relevant Rayleigh damping parameters were incorporated into the model: for all non-osseous soft tissue structures, Rayleigh coefficients α = 0 s^−1^ and β = 0.75 × 10^−4^ s were applied (De Greef et al., 2015; 2017). The distal insertion faces of the AML, LML, PIL, SAL, and SM were defined as fixed boundary conditions. The circumferential boundary of the tympanic membrane was also fully constrained to simulate the physiological supporting effect of the external auditory canal bony annulus. Contact interfaces between anatomical components were modeled using multipoint constraint (MPC) bonded conditions to simulate rigid coupling. The ear canal lumen was modeled as an air-filled domain, and a uniform 100 sound pressure (2 Pa) excitation was applied across the 100 Hz–10 kHz frequency range at the lateral canal entrance to simulate physiological auditory stimulation. A fluid-structure coupling interface was defined at the air-tympanic membrane boundary. Perilymph loading on the stapes footplate was simulated using a distributed spring load with a lumped constant of 0.06 N/mm (Thompson et al., 2023). The vibratory velocity at the central node of the stapes footplate was extracted to quantify the vibratory conduction function of the middle ear.

**TABLE 1:** Material mechanics parameters related to finite element models.

Structure	*E*(MPa)	*ρ*(kg/m^3^)
TM tensa	35	1,200
TM flaccida	10	1,200
Malleus head	14,100	2,550
Malleus neck	14,100	4,530
Malleus handle	14,100	3,700
Incus body	14,100	2,360
Incus short process	14,100	2,260
Incus long process	14,100	5,080
Stapes	14,100	2,200
IMJ	14,100	3,200
ISJ	0.6	1,200
TTM	5	1,500
AML	21	1,500
LML	6.7	1,500
PIL	6.5	1,500
SAL	1.1	200
SM	0.38	1,500
Ti-6Al-4V	96,000	4,620
PEEK	3,800	1,290

### 2.3 FEA model of PORP-implanted middle ear

Using the validated intact middle ear finite element model as a baseline, the malleus, incus, IMJ, ISJ, tensor tympani tendon, AML, LML, and PIL were surgically resected to generate a TM-PORP reconstructed middle ear model (Figure 3). Contact interfaces between the PORP and tympanic membrane/stapes head were modeled with rough contact to approximate *in vivo* coupling conditions. A 0.05 mm interference fit is applied along the contact surface normal direction to achieve press-fit engagement between the PEEK connector and the stapes head. Hearing reconstruction performance was evaluated using the vibratory velocity frequency response curve of the stapes footplate. The assigned elastic modulus (E) and density (ρ) values for titanium alloy and medical-grade PEEK used in the FEA are listed in Table 1. The yield stress of Ti-6Al-4V is 1,098 MPa. The stapes footplate velocity frequency response extracted from the PORP-implanted harmonic analysis was directly compared against the baseline intact middle ear response to quantify hearing reconstruction fidelity. Total prosthesis length was systematically adjusted across the full 2.3–3.5 mm range to evaluate the relationship between intraoperative length calibration and vibratory conduction performance. For each tested length configuration, the position and orientation of the PEEK stapes bowl and its coupling to the native stapes capitulum remain fully unchanged and locked relative to the baseline intact middle ear anatomical position; the accompanying lateral offset produced by length adjustment is accommodated by rigid translation of the stapes bone. The tympanic-side PEEK disc maintains consistent surface contact orientation and fixed contact location on the medial surface of the tympanic membrane across all length configurations, so that the vibratory load transmission pathway between the prosthesis and tympanic membrane is kept identical across all test groups. This standardized alignment approach minimizes geometric variability across test conditions. Consequently, observed differences in sound transmission primarily reflect the mechanical response of the prosthesis at different length settings. The full 3D CAD geometry of the PORP prosthesis was provided by Suzhou Zhenyi Medical Technology Co., Ltd.

**FIGURE 3:**
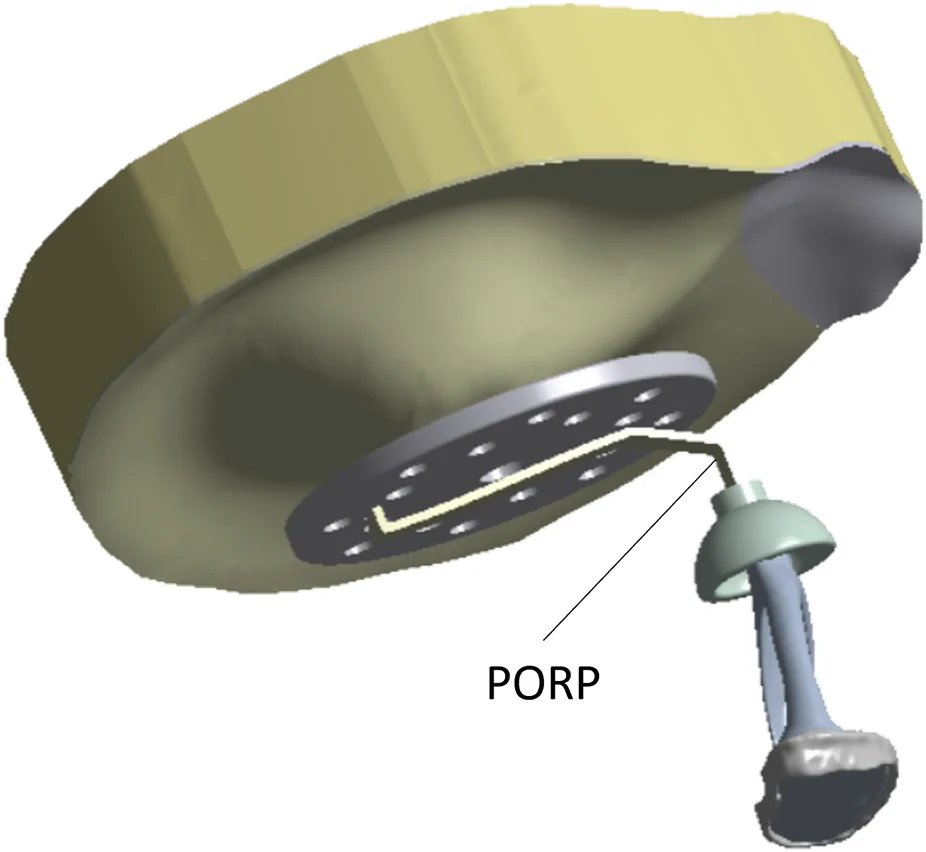
Finite element model of PORP-implanted middle ear for sound conduction simulation.

## 3 Results

### 3.1 Stress distribution of PORP at different adjusted lengths

Figure 4 presents the von Mises stress distribution within the novel adjustable-length PORP prosthesis across the evaluated length range (2.3–3.5 mm). Peak stress concentrations were consistently localized to the curved bending surface of the central titanium connecting rod. Figure 5 plots the relationship between maximum von Mises stress and adjusted prosthesis length. Across the 2.3–3.5 mm range, maximum von Mises stress remained relatively stable near 1,098 MPa, which is the yield stress of Ti-6Al-4V. These results indicate that the connecting rod undergoes plastic deformation during adjustment, with negligible elastic recovery after force removal. This ensures stable retention of the calibrated position post-implantation.

**FIGURE 4:**
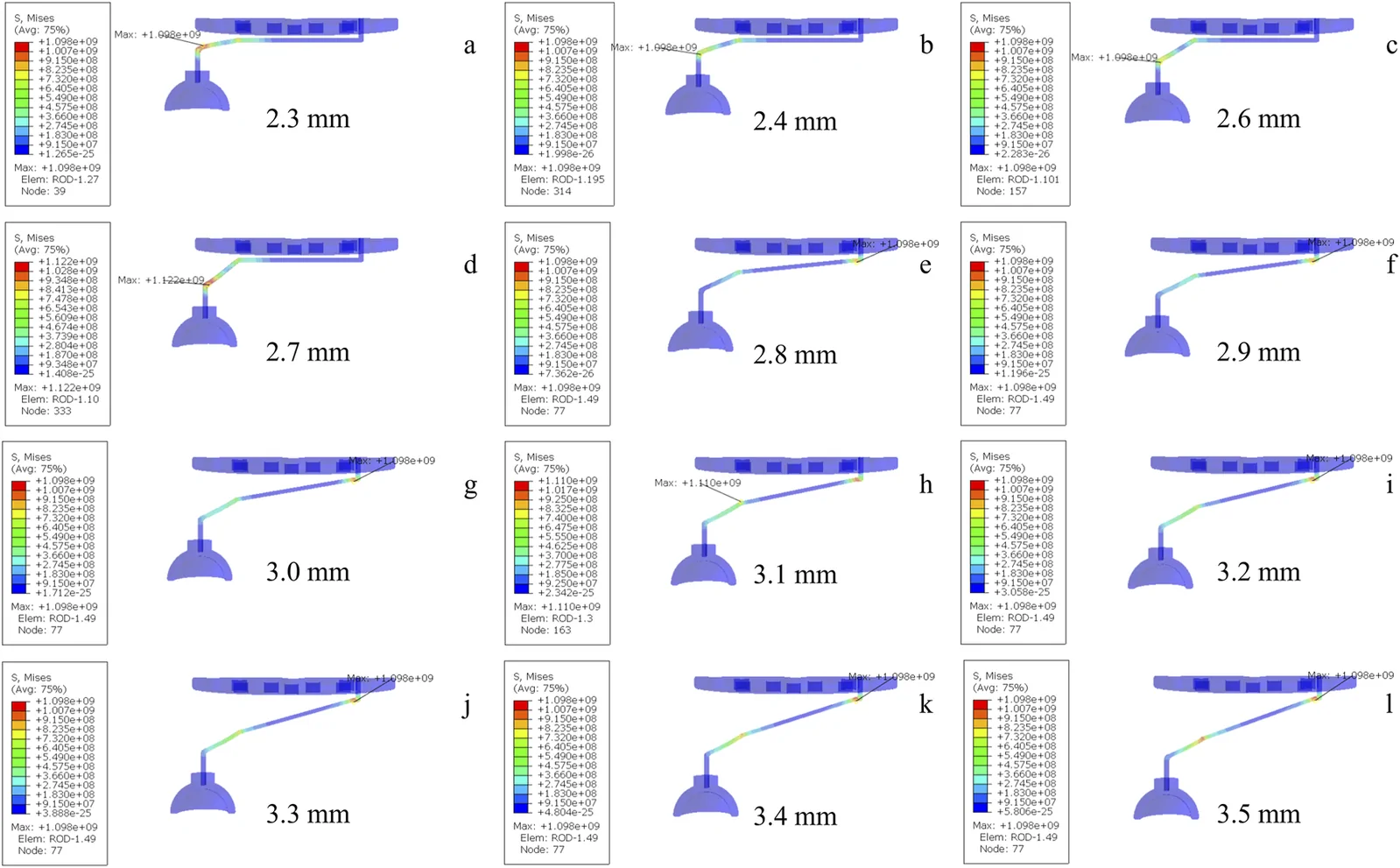
Contour plots of von Mises stress distribution in the PORP across the full 2.3–3.5 mm length adjustment range (unit: Pa) **(a–l)**: 2.3–3.5 mm.

**FIGURE 5:**
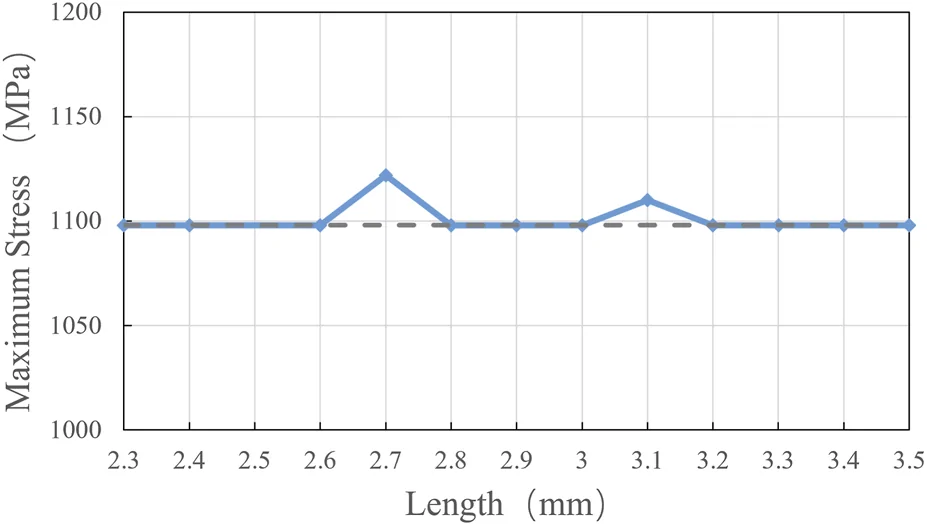
Maximum von Mises stress variation of the titanium alloy connecting rod across the adjusted PORP length range.

### 3.2 Hearing reconstruction performance across calibrated PORP lengths

Figure 6 presents the stress distribution across the reconstructed middle ear system following implantation of the 3.5 mm PORP configuration. The full-system stress distribution (Figure 6a) demonstrates maximum stresses localized to the prosthesis-tympanic membrane interface. The maximum von Mises stress on the tympanic membrane (Figure 6b) was 0.89 MPa. Peak stress on the native stapes superstructure (Figure 6c) was only 0.01 MPa. The peak von Mises stress within the PORP component (Figure 6d) was 0.33 MPa. Peak stress on the tympanic-side PEEK disc (Figure 6e) was 0.33 MPa. Finally, peak stress on the PEEK stapes bowl (Figure 6f) was 0.008 MPa.

**FIGURE 6:**
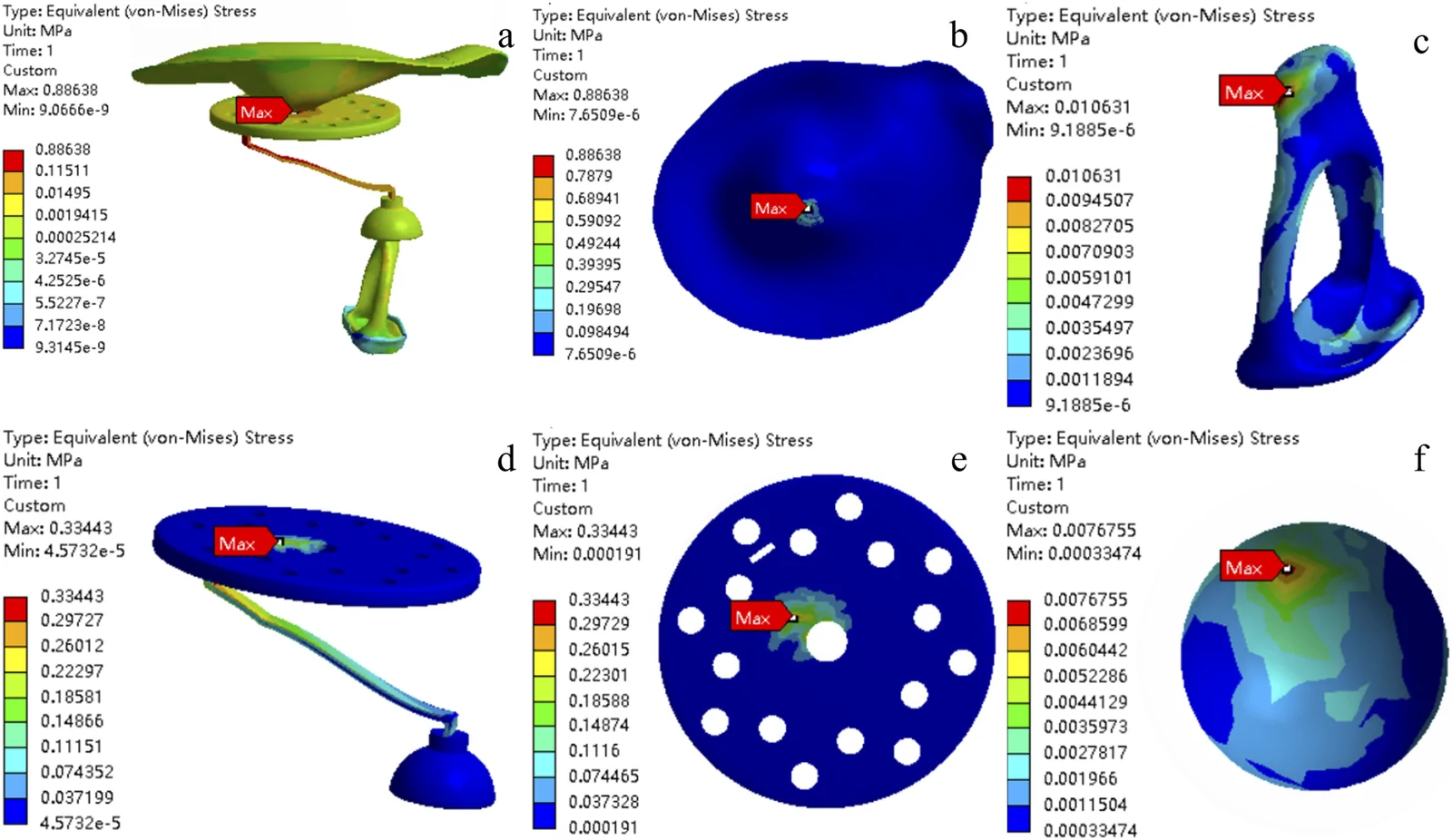
Full-system stress distribution of the reconstructed middle ear following PORP implantation at 3.5 mm length. **(a)** Global middle ear system stress; **(b)** Tympanic membrane stress; **(c)** Native stapes superstructure stress; **(d)** PORP prosthesis stress; **(e)** Tympanic-side PEEK disc stress; **(f)** PEEK stapes connector stress.

Figure 7 plots the stapes footplate vibratory velocity and phase frequency response curves across all calibrated PORP lengths (2.3–3.5 mm). The intact native middle ear baseline response (black curve) demonstrates the well-documented physiological behavior: stapes footplate vibratory velocity increases gradually with frequency across the 100–750 Hz range, forms a broad plateau across 750–3,000 Hz, and decays rapidly with increasing frequency above 3 kHz. The intact-model results align with published laser Doppler vibrometry measurements and finite element simulations (Thompson et al., 2023; Sokołowski et al., 2026), supporting model validity. Figure 7a shows the velocity-frequency response curves following PORP implantation at lengths shorter than the baseline 2.5 mm reference value, while Figure 7c presents velocity-frequency response curves at lengths longer than 2.5 mm. The overall shape of the stapes footplate velocity transfer function after PORP implantation remained highly consistent with the intact native middle ear response across all valid length configurations. The phase frequency response curves (Figures 7b,d) demonstrate that the adjustable PORP preserves the temporal characteristics of sound transmission. The maximum phase deviation relative to the intact middle ear baseline remains within 50° across the evaluated spectrum (100 Hz–10 kHz), indicating that the deformable titanium connecting rod does not introduce significant temporal delays or phase distortions that would compromise the temporal fidelity essential for accurate auditory perception.

**FIGURE 7:**
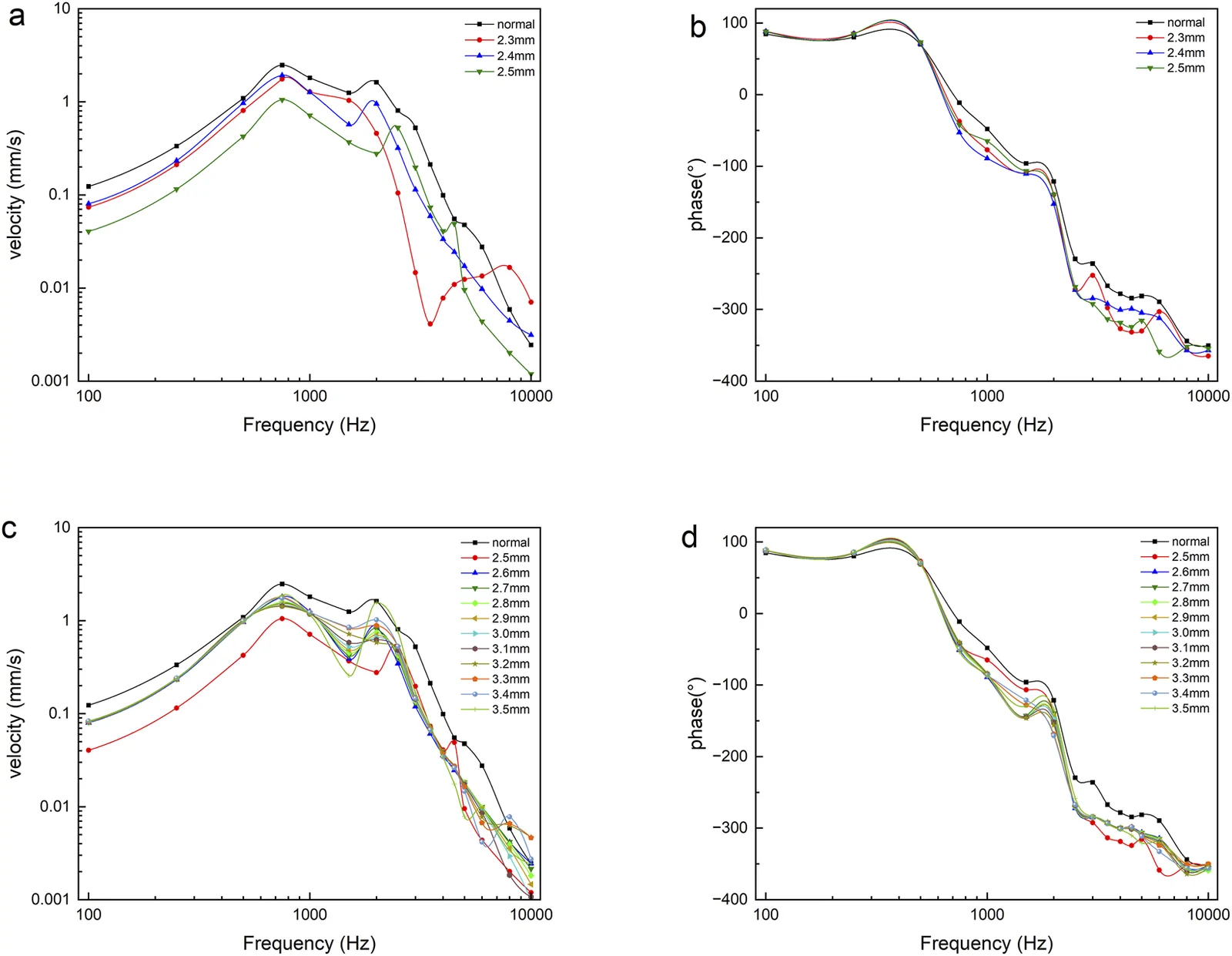
Stapes footplate vibratory velocity and phase frequency response curves across all calibrated PORP lengths. **(a,b)** 2.3–2.5 mm short-length range; **(c,d)** 2.5–3.5 mm functional length range.

To enable direct, clinically interpretable quantification of hearing reconstruction fidelity, hearing loss relative to the intact middle ear was defined as: ( $\text{hearing loss}=20\;\mathrm{lg}\frac{v_{PORP}}{v_{normal}}$ ) where $v_{PORP}$ is the vibratory velocity extracted at the central stapes footplate node in the PORP-implanted finite element model, and $v_{normal}$ is the corresponding velocity from the validated intact middle ear baseline model. Hearing loss values across all tested PORP lengths are summarized in Table 2. A negative value indicates reduced stapes velocity (i.e., hearing loss relative to the intact ear), while a positive value indicates increased velocity relative to the intact baseline. Peak hearing loss varied across frequency regions depending on prosthesis length configuration. For shorter lengths (2.4–3.1 mm), maximum hearing loss was predominantly localized to the 3,000–3,500 Hz frequency region, corresponding to the natural resonance frequency of the human middle ear ossicular chain. For longer configurations (3.2–3.5 mm), peak hearing loss shifted to higher frequencies (5,000–6,000 Hz). Across the 2.4–3.5 mm length range, maximum hearing loss remained below 17 dB, demonstrating sound transmission performance comparable to the native ossicular chain. At the minimum tested length of 2.3 mm, maximum hearing loss increased to 34 dB.

**TABLE 2:** Frequency-specific hearing loss (dB) at different PORP lengths.

Frequency/Hz	PORP length/mm												
	2.3	2.4	2.5	2.6	2.7	2.8	2.9	3.0	3.1	3.2	3.3	3.4	3.5
100 Hz	−4.4	−3.7	−9.7	−3.7	−3.7	−3.6	−3.6	−3.6	−3.5	−3.5	−3.4	−3.4	−3.4
250 Hz	−4.0	−3.1	−9.3	−3.2	−3.1	−3.1	−3.0	−3.0	−3.0	−2.9	−2.9	−2.8	−2.9
500 Hz	−2.6	−1.0	−8.2	−1.0	−0.8	−0.8	−0.8	−0.7	−0.6	−0.6	−0.9	−0.8	−1.0
750 Hz	−3.0	−2.2	−7.4	−2.8	−4.1	−3.9	−4.3	−4.5	−4.7	−4.8	−2.9	−3.0	−2.4
1,000 Hz	−3.0	−3.1	−8.1	−3.2	−3.5	−3.6	−3.6	−3.7	−3.7	−3.7	−3.3	−3.3	−3.9
1,500 Hz	−1.6	−6.8	−10.6	−10.1	−9.5	−8.9	−8.2	−7.5	−6.6	−4.8	−3.5	−3.3	−13.7
2,000 Hz	−11.0	−4.6	−15.4	−5.6	−6.0	−6.8	−7.3	−7.8	−8.3	−8.9	−5.2	−4.0	−0.3
2,500 Hz	−17.7	−8.1	−3.7	−7.4	−6.0	−5.6	−5.2	−4.8	−4.5	−3.7	−3.7	−3.7	−0.4
3,000 Hz	−31.1	−13.2	−8.5	−12.8	−12.0	−11.7	−11.5	−11.3	−11.2	−10.9	−11.0	−11.1	−9.9
3,500 Hz	−34.3	−11.1	−9.2	−10.8	−10.2	−10.0	−9.9	−9.8	−9.7	−9.6	−9.8	−10.0	−9.1
4,000 Hz	−22.1	−9.4	−7.7	−8.8	−8.5	−8.2	−8.1	−8.2	−8.3	−8.3	−8.6	−9.1	−9.3
4,500 Hz	−14.1	−7.1	−1.0	−7.0	−6.3	−6.3	−6.3	−6.3	−6.3	−6.3	−6.2	−6.5	−9.9
5,000 Hz	−11.7	−8.9	−14.0	−8.8	−8.3	−8.3	−8.4	−8.5	−8.7	−9.0	−9.4	−10.2	−15.7
6,000 Hz	−6.2	−9.0	−16.0	−9.2	−8.9	−9.0	−9.2	−9.6	−10.1	−11.0	−12.3	−16.4	−9.3
8,000 Hz	9.0	−2.4	−9.3	−3.0	−2.9	−3.5	−4.4	−6.0	−10.1	0.6	8.6	2.5	−4.8
10,000 Hz	9.2	2.1	−6.3	0.0	−1.2	−2.6	−4.5	−7.8	−7.2	5.6	5.5	0.9	0.0

## 4 Discussion

This study presents a novel titanium alloy-PEEK hybrid adjustable-length partial ossicular replacement prosthesis, and systematically evaluates its hearing reconstruction performance using a validated anatomically accurate middle ear finite element model. The results demonstrate that the proposed prosthesis preserves stable stapes footplate vibratory transmission across a clinically relevant range of length calibrations.

Finite element analysis (FEA) is widely used for design optimization and biomechanical evaluation in ossicular reconstruction research (Jiang et al., 2025; Mohseni-Dargah et al., 2025; Lähde et al., 2026; Muyshondt et al., 2026; Zhou et al., 2026). In this study, hearing reconstruction performance was quantified by directly comparing stapes footplate vibratory velocity responses between the intact and PORP-reconstructed FEA models. The intact middle ear stapes footplate velocity frequency response generated in this study demonstrates near-quantitative agreement with experimental laser Doppler vibrometry measurements and validated finite element results reported independently in the literature (Thompson et al., 2023; Sokołowski et al., 2026), supporting the model’s utility for evaluating PORP vibro-acoustic performance.

Current clinically available “adjustable-length” PORP designs achieve length modification exclusively via irreversible manual intraoperative trimming. Surgeons frequently find that the device length after initial trimming remains suboptimal, necessitating a second trimming step that prolongs operative time, increases surgical risk, and can result in complete device wastage if the prosthesis is inadvertently cut too short. The proposed novel PORP design achieves continuous length adjustment via controlled plastic deformation of the bent titanium connecting rod. Structural analysis confirms that the titanium alloy connecting rod undergoes plastic deformation across the 2.3–3.5 mm length range, with maximum von Mises stress of about 1,098 MPa, which is the yield strength of the Ti-6Al-4V. This indicates negligible elastic recovery following force removal, ensuring stable retention of the calibrated position.

Hearing reconstruction performance evaluation demonstrates that the overall shape of the stapes footplate velocity frequency response after PORP implantation closely mirrors that of the intact native middle ear. Peak hearing loss varied across frequency regions depending on prosthesis length configuration. For shorter lengths (2.4–3.1 mm), maximum hearing loss was predominantly localized to the 3,000–3,500 Hz frequency region, corresponding to the natural resonance frequency of the human middle ear ossicular chain. For longer configurations (3.2–3.5 mm), peak hearing loss shifted to higher frequencies (5,000–6,000 Hz). The prosthesis maintains a stable stapes footplate velocity transfer function (hearing loss <17 dB) across the length calibration range of 2.4–3.5 mm. Conventional clinical benchmarks define PORP implantation as functionally successful if the postoperative ABG is <20 dB (Kraus, 1993; Nguyen et al., 2021). The predicted maximum hearing loss of 17 dB falls within the conventional clinical success criterion (ABG ≤20 dB), suggesting potential for adequate hearing reconstruction.

For contemporary ossicular prostheses, titanium alloy remains the dominant clinical material due to its high intrinsic strength and well-characterized long-term biocompatibility (Govil et al., 2017; Faramarzi et al., 2023). However, because the elastic modulus of titanium alloy is nearly an order of magnitude higher than that of native ossicular bone, pure titanium prostheses inevitably induce clinically significant stress shielding (Wang et al., 2023; Sergienko et al., 2025), driving periprosthetic bone resorption, increasing risks of prosthesis extrusion and aseptic loosening, and eventually contributing to long-term hearing reconstruction failure (Zheng et al., 2026a; 2026b). An ossicular prosthesis material with mechanical properties closely matched to native ossicular tissue is expected to materially reduce these established complication risks. PEEK has seen rapidly expanding clinical adoption as an orthopedic bone substitute material due to its excellent biocompatibility and elastic modulus closely matching cortical bone (Ferris and Prendergast, 2000; Wang et al., 2022; Zheng et al., 2023), though no large-scale clinical series evaluating PEEK-based ossicular prostheses have been published to date. The finite element results presented here demonstrate that peak stress on the tympanic membrane after PORP implantation is only 0.89 MPa. These findings suggest reduced mechanical stress on the tympanic membrane compared to titanium prostheses, potentially lowering complication risks. However, experimental validation through cadaveric studies and animal trials is required. Peak von Mises stress on the native stapes superstructure is only 0.01 MPa, which matches closely with the peak stress measured on the PEEK stapes connector (0.008 MPa), confirming that the design effectively minimizes stress mismatch and reduces stress shielding-driven prosthesis extrusion risk. However, due to the relatively low inherent elastic modulus of PEEK, *in vivo* osseointegration performance and long-term stability of vibro-acoustic transmission after chronic implantation remain to be experimentally validated. Future material optimization could consider higher-modulus fiber-reinforced PEEK composites (Chen et al., 2022), or emerging low-modulus β-titanium alloy formulations (Solano-Orrala et al., 2026) as additional pathways to further refine PORP mechanical performance.

We acknowledge several limitations of the present study. First, the analysis is based on a single middle ear geometry derived from one cadaveric temporal bone. Systematic sensitivity analyses examining the effects of prosthesis positioning, orientation, and contact location were not performed. Second, the present study only evaluated the direct PEEK–tympanic membrane contact configuration without cartilage interposition; additional comparative simulations will be required to extend these findings to clinical workflows where surgeons elect to place a cartilage graft between the PEEK disc and tympanic membrane. Third, the piston-component velocity metric does not capture rotational motion components of the stapes footplate that become more prominent at frequencies above 4 kHz; future work incorporating full 3D footplate motion field analysis will further refine the simulation accuracy. Fourth, long-term osseointegration and vibro-acoustic stability of PEEK implants require validation through animal studies and clinical trials. Further iterative optimization of the PORP dimensional parameters could also extend the functional length adjustment range and improve high-frequency sound transmission fidelity.

## Data Availability

The original contributions presented in the study are included in the article/supplementary material, further inquiries can be directed to the corresponding author.

## Footnotes

1
https://www.uantwerpen.be/en/rg/bimef/downloads/
